# Brief Interventions for Alcohol Problems: Factors That Facilitate Implementation

**Published:** 2004

**Authors:** Anne Moyer, John W. Finney

**Affiliations:** Anne Moyer, Ph.D., is an assistant professor of psychology at the State University of New York at Stony Brook, Stony Brook, New York. John W. Finney, Ph.D., is director of the Health Services Research & Development Center for Health Care Evaluation, VA Palo Alto Health Care System, Palo Alto, California

**Keywords:** harmful drinking, hazardous drinking, risk assessment, intervention (persuasion to treatment), prevention, intervention process and procedures, brief intervention, motivational interviewing, counseling, peer counseling, normative education, computer-assisted instruction, primary care facility, emergency room, trauma center

## Abstract

Brief interventions are gaining favor as a means of addressing the problems associated with hazardous and harmful drinking. Brief interventions commonly target people whose levels or patterns of use are not diagnosable as alcohol abuse or dependence. These interventions usually are delivered by professionals who do not specialize in alcoholism treatment, and they include treatment elements designed to encourage people to alter their alcohol use without creating resistance. As evidence mounts regarding the efficacy of these interventions, attention has turned to implementing them successfully. New modes of delivery, such as via computers, the Internet, and interactive multimedia presentations, may help to surmount some of the challenges of wide dissemination, such as strains on expertise, time, and resources.

As those in the alcohol field recognize that the problems associated with drinking begin at alcohol consumption levels much lower than those previously thought to warrant treatment, brief interventions have become an important tool in the intervention armamentarium. In addition, the importance of secondary prevention has become more widely acknowledged as research evidence has accumulated regarding the reduction of health care and social costs that may be achieved with brief interventions.

## Features of Brief Interventions

Brief interventions typically emphasize reducing a person’s alcohol consumption to nonhazardous levels and eliminating binge drinking rather than insisting that the person abstain from drinking, although abstinence also may be a welcomed goal. A common aim is to intervene early and target people whose levels of drinking or patterns of use would be considered hazardous or harmful^1^ and to reduce problems associated with drinking, such as alcohol-related medical problems, injuries, domestic violence, motor vehicle crashes, arrests, or damage to a fetus. Accordingly, brief interventions do not usually target people whose levels or patterns of drinking meet diagnostic criteria for alcohol abuse or alcohol dependence, although they sometimes may be used to motivate an abusing or dependent drinker to seek more intensive alcohol-related treatment or as a first stage in a “stepped” care model, in which more intensive/extensive treatment would be provided if brief intervention failed.

People drinking at levels thought to be suitable for brief interventions often are identified at their primary care facility when they are screened during a routine health care visit, or at the hospital during a stay on a medical unit for a different condition. Alternatively, people who could benefit from brief interventions can be identified by an event precipitated by their problematic alcohol use (e.g., an emergency room visit for alcohol-related injuries or an arrest for driving while intoxicated), or they may be in situations in which drinking is particularly harmful (e.g., while pregnant). Clinicians and others who are in the position to give brief interventions could capitalize on these occasions when people may be particularly receptive to advice to alter their drinking. (See the textbox for a summary of settings in which people can be assessed for alcohol problems.)

**Settings in Which People Could Be Assessed for Alcohol Problems**

- Primary care offices, when patients are seen for routine visits
- Hospitals, when patients are treated for conditions that are not alcohol related
- Emergency rooms or trauma centers, when accident victims come for treatment for alcohol-related injuries, such as car crashes, falls, or fights
- Police stations, when drivers are arrested for driving while intoxicated
- OB-GYN offices, when pregnant women come for prenatal care.

Often, a nonspecialist authority figure whom the patient may already trust or feel comfortable being treated by—such as a physician, a nurse or physician’s assistant in a primary care setting, or a nurse or physician’s assistant on a medical unit—delivers the brief intervention. (The sidebar “Implementing and Disseminating Brief Interventions” discusses the effect of professionals’ attitudes toward delivering brief interventions.) Brief interventions usually involve individualized feedback and counseling based on an assessment that the patient is at risk for harmful drinking. Such feedback in itself may encourage some hazardous or harmful drinkers to reduce their alcohol intake.

**Implementing and Disseminating Brief Interventions**

Medical, mental health, and even legal settings offer numerous opportunities for people working in these environments to recognize and intervene productively with people who have alcohol consumption patterns that are causing negative consequences but have not yet led to alcohol dependence. Although clients may be receptive, brief interventions seldom are used in these settings. Accordingly, some researchers have begun to investigate the attitudes of professionals that could influence whether they provide brief interventions, as revealed by the two studies with primary care providers described here.

Using focus groups of 18 general practitioners and 19 nurses, Aalto and colleagues (2003*a*) identified some of the potential challenges to carrying out brief interventions in primary care settings. Finnish general practitioners and nurses had the following reactions:

- They were uncertain about what constituted hazardous or harmful drinking, how it could be distinguished from alcohol dependence, and how to identify it.
- They felt awkward about initiating conversations about alcohol without a legitimate clinical reason.
- They felt they had insufficient time, knowledge, and expertise to deliver brief interventions.

The authors also noted that some primary care professionals could be uncomfortable using the nonauthoritarian approach typically employed in brief intervention studies.

To address primary care clinicians’ belief that they lacked expertise to discuss alcohol-related topics with their patients, researchers examined the occurrence and duration of alcohol-related discussions before and after clinicians received training in brief interventions (Vinson et al. 2000). This training consisted of a videotaped lecture and an accompanying physician’s guide to helping patients with alcohol problems. After clinicians watched the video, the investigators called them to discuss any concerns or any barriers they had to delivering the interventions. The main finding was that subsequent to the training, the alcohol-related discussions the practitioners had with patients were significantly longer. However, even when practitioners knew that patients had positive screening results, only 26 percent of these discussions lasted longer than 4 minutes, which is shorter than the 5- to 15-minute intervention typically tested in research.

Other implementation research has used other approaches—including developing clinical practice guidelines, training primary health care professionals, and raising the public’s awareness of the concept of hazardous drinking—with modest results (Aalto et al. 2003*b*). Proponents of a public health model of clinical preventive services have emphasized the importance of making changes in communities in which brief interventions are provided so that the majority of the population understands the health risks associated with excessive drinking and supports the application of brief interventions, potentially via social marketing (for more on this public health model, see Babor and Higgins-Biddle 2000).

—Anne Moyer and John W. Finney

ReferencesAaltoMPekuriPSeppaKObstacles to carrying out brief interventions for heavy drinkers in primary care: A focus group studyDrug and Alcohol Review221691732003a1285090310.1080/09595230100100606AaltoMPekuriPSeppaKPrimary health care professionals’ activity in intervening in patients’ alcohol drinking during a 3-year brief implementation projectDrug and Alcohol Dependence699142003b1253606110.1016/s0376-8716(02)00228-4BaborTFHiggins-BiddleJCAlcohol screening and brief intervention: Dissemination strategies for medical practice and public healthAddiction9567768620001088504210.1046/j.1360-0443.2000.9556773.xVinsonDCElderNWernerJJAlcohol-related discussions in primary care: A report from ASPNJournal of Family Practice492833200010678337

Brief interventions also commonly use client-centered approaches, which are designed to motivate people who may resist suggestions to moderate their alcohol intake or may help to reach people who do not believe they are drinking in a harmful or hazardous way. Motivational interviewing (Miller and Rollnick 1991) is an important client-centered technique that uses empathy and warmth rather than confrontation to encourage people to decide for themselves to change. In addition to offering encouragement or advice to change, clinicians providing brief interventions typically help their patients establish goals and provide specific skill-building strategies they can use in modifying their drinking behavior. Clinicians can include supplemental materials, such as pamphlets, manuals, or workbooks, to help convey and reinforce these strategies. After this initial contact, clinicians can provide followup with additional assessment and advice to clarify and bolster the strategies and goals. If a brief intervention is not successful in motivating a patient to reduce alcohol consumption, the clinician then can recommend that the person seek more extensive treatment.

Receiving an intervention or materials in a primary care setting may be particularly appealing to patients who could be engaged in harmful or hazardous drinking. They might object to the potentially embarrassing, stigmatizing, or inconvenient features of entering an alcoholism treatment program, consulting with an addiction specialist, or taking time away from work or family responsibilities. Brief interventions also are useful because of their lower health care costs compared with more formal specialist alcoholism treatment.

## Evidence Basis for Effective Brief Interventions

Various researchers have studied brief interventions to identify approaches that can be used successfully with a variety of target audiences seen in different settings. The studies described in the following sections focus on different methods for delivering effective brief interventions.

### Brief Interventions Provided in Nonmedical Settings

In their study of effective methods to use with college students, Collins and colleagues (2002) used a brief, nonconfrontational motivational intervention that focused on encouraging students to change their drinking behavior by showing them the discrepancy between how they viewed their own behavior and what they actually were doing. The researchers mailed each participating student an individual report that included information, based on the student’s self-report, indicating how much and how frequently the student drank, how often he or she engaged in heavy drinking episodes, as well as the student’s typical and peak blood alcohol levels and his or her alcohol-related problems. In addition, the report provided data on the norms for these variables among peers at the national and university levels. This personalized normative feedback was intended to make participants aware of the level and consequences of their drinking and how these compared with the drinking behavior of others to whom they could relate. In keeping with the investigators’ motivational approach, which explicitly avoided fostering resistance, the reports did not tell the students that they were considered at risk; they were left to draw their own conclusions from the feedback. A control group was mailed a psychoeducational brochure about alcohol use.

Short-term results were promising: At 6-week followup, students who received the mailed feedback reported having fewer heavy-drinking episodes and consuming fewer drinks during their heaviest drinking week than did students in the control group. However, the significant effects seen at 6 weeks were no longer evident at 6 months. Although this type of mailed feedback is low in cost and requires few resources, the researchers suggested that for the effects to last beyond the short term, some form of booster contact may be necessary.

### Brief Interventions Delivered in Primary Care Settings

The longer term effects of an intervention that included booster or followup contact after the initial brief intervention were investigated by Fleming and colleagues (2002) in a randomized trial examining brief advice sessions delivered by physicians to patients identified as problem drinkers. Potential participants were screened during a routine office visit with an instrument that posed questions about several health indicators in addition to alcohol use. Both intervention and control group participants received a general health booklet on behaviors such as exercise, nutrition, seat belt use, safe sex, and alcohol and other drug use. The intervention consisted of two 15-minute doctor visits 1 month apart, which focused on the prevalence and effects of problem drinking, followed up with two telephone calls by nurses 2 weeks later. Those in the intervention group also received a worksheet about drinking cues, cards on which to keep a drinking diary, and a drinking agreement.

This study overcame many of the limitations prevalent in this literature by doing the following:

- Recruiting an adequately sized sample (*N* = 774)
- Having physicians work from a scripted workbook
- Striving to maximize followup (e.g., by paying participants $110 for completing research procedures, collecting phone numbers of friends and family members)
- Embedding alcohol-related outcome assessments in the context of other health-related questions (to prevent control group participants from recognizing the intent of the study and, as a result, attempting to reduce their alcohol use)
- Keeping physicians unaware of the patients in their practice who were in the control group (to avoid the temptation to intervene with them)
- Relying on records rather than self-reports for legal and motor vehicle incident information
- Conducting conservative statistical analyses by assuming a poor outcome for participants who were randomized to treatment but then did not participate in treatment and for those lost to followup.

At 6-, 12-, 24-, 36-, and 48-month followup points, the brief intervention group had significantly better outcomes than the control group in terms of reductions in the following areas: number of drinks per week, number of binge drinking episodes during the prior 30 days, percentage of heavier drinkers, percentage of people who reported binge drinking episodes during the prior 30 days, emergency department visits, days of hospitalization, and arrests for controlled substance or liquor violations. This study shows that providing patients with followup sessions after their initial intervention resulted in long-lasting improvement.

Ockene and colleagues (1999) used nurse practitioners in addition to physicians to deliver a counseling intervention because nurse practitioners are educated to provide preventive care, counseling, and patient education. A 5- to 10-minute patient-centered, collaborative counseling session was delivered by practitioners who had received 2.5 hours of training that emphasized questioning and feedback skills. Office support staff assisted in implementing the intervention by reminding providers of the steps in the counseling protocol, giving a summary of the patient’s alcohol history, and handing out patient education materials. Compared with patients who received usual care, at 6 months the intervention group had a significantly more pronounced reduction in the number of drinks consumed per week but not in the number of binge drinking episodes per month. Similarly, significantly more intervention group participants were drinking at safe levels, but there were no group differences in the number of binge drinkers who stopped binge drinking completely. The findings of this study are encouraging because they show that using nurse practitioners to deliver brief interventions can decrease the burden on physicians (Ockene et al. 1999).

### Brief Interventions Implemented in Emergency Departments and Trauma Centers

Alcohol plays an important role in causing many traumatic injuries. For this reason, several authors have suggested that people treated in emergency rooms and in trauma centers should be screened routinely for alcohol misuse and that those who screen positive should receive some kind of intervention. The following studies show how some of the components discussed above, such as followup sessions and audience-targeted interventions, can be used in emergency departments and trauma centers. (Also see the article “Screening and Brief Intervention in the Emergency Department” by D’Onofrio and Degutis, in the companion issue.)

Gentilello and colleagues (1999) randomized injured patients treated in a trauma center who screened positive for an alcohol problem to receive a brief motivational intervention or standard care. The motivational intervention included providing feedback (e.g., telling patients their level of intoxication at admission and its connection to their injury), emphasizing each patient’s responsibility for change, offering respectful advice, suggesting a menu of tools and strategies for change, using an empathetic style of delivery, and encouraging feelings of self-efficacy. The brief intervention was intended to fit within the constraints of a trauma center; it lasted about 30 minutes, was delivered on or near the day of discharge by a psychologist trained in brief interventions, and was followed by a handwritten summary sent to the patient about 1 month later. The primary outcome variable, trauma recurrence, occurred significantly less often 12 months later among those in the intervention group relative to those in the standard care group. The intervention was most effective for patients who were not married and not employed—presumably those who were least socially stable and most in need of assistance.

In a study by Longabaugh and colleagues (2001), patients receiving care at an emergency department were offered a brief intervention, or a brief intervention plus a booster session 7 to 10 days later, or standard emergency department care. Patients were asked to participate if they screened positive for hazardous or harmful drinking. The booster session was meant to address the time limitations of an emergency visit and the distractions (such as pain, treatment for injuries, waiting family members, or the influence of alcohol) that could reduce the participants’ ability to benefit from the intervention.

During the brief intervention, which lasted 40 to 60 minutes, a trained interventionist used an empathetic, nonconfrontational, motivational enhancement approach. Each discussion centered on exploring the possible connection between alcohol use and the patient’s injury, how the patient’s level of drinking compared with a national sample, and the pros and cons of drinking. For patients who wanted to change their behaviors, the interventionist drew up a change plan worksheet that recorded their reasons for wanting to change, the steps they planned to take, ways others could help them, potential difficulties, and what they could do to evaluate their progress. For the intervention group receiving the booster session, this second encounter explored experiences patients had had since discharge and provided an opportunity for them to revise the change plan based on these experiences. Booster group participants also were given additional information about their alcohol use based on a questionnaire about alcohol expectancies that they completed at the prior session.

Results at 1-year followup indicated that those receiving the booster session along with the brief intervention, but not the brief intervention alone, had reduced alcohol-related negative consequences and alcohol-related injuries relative to those who received standard emergency department care. However, the brief intervention plus the booster session was not more likely to reduce heavy drinking or injuries unrelated to alcohol use. This finding could be explained by the fact that the intervention was specifically focused on alcohol-related problems such as injuries resulting from alcohol use, not on drinking or reckless behavior unrelated to alcohol use that also could lead to injury. Surprisingly, regardless of whether alcohol was involved in the injury that brought patients to the emergency department, the brief intervention plus the booster session was effective in reducing alcohol-related negative consequences. The authors concluded that any injury which brings heavy drinkers to the emergency department can provide an opportunity to offer interventions, but motivating patients to participate in a followup visit is crucial.

Monti and colleagues (1999) investigated a brief intervention directed at adolescents who, during an ED visit, tested positive for alcohol use. Patients had to pass a mental status examination (an examination certifying that they were not too alcohol impaired to consent to and participate in the study) before participating. One group received standard care, which included getting a handout on avoiding drinking and driving and a listing of local treatment agencies; the other group received a 35- to 40-minute motivational intervention. Based on the principles of motivational interviewing, the interventionist discussed with the patients the circumstances of the injury that brought them to the emergency department and the advantages and disadvantages of drinking, provided personalized feedback based on the information from their alcohol use assessment, developed plans for the future, and helped the adolescents in establishing goals.

These researchers found a reduction in alcohol consumption in both the intervention and control groups (the latter group possibly was affected by the injury that brought them to the emergency department, which itself could serve as an intervention effect). However, compared with the control group, the intervention group also engaged in fewer problematic behaviors, such as driving after drinking, and had fewer moving violations, alcohol-related injuries, and alcohol-related problems. Although the intervention did not involve a followup treatment session, the authors noted difficulties in recruiting participants because the adolescents were eager to be discharged from the hospital once their mental status had cleared.

As noted in the Longabaugh study above, patients being treated in emergency departments may be too distracted for a variety of reasons to benefit fully from the brief interventions they receive during those visits. In addition, emergency department patients typically leave quickly after being treated for the injury that brought them to that facility. Providers delivering brief interventions to patients who have been admitted to the hospital for longer term care may capitalize on the less distracting environment, possibly eliminating the need for patients to return for additional sessions. Blondell and colleagues (2001) investigated brief interventions delivered to patients hospitalized for alcohol-related injuries. Specifically, they tested whether the combination of an intervention delivered by peers (recovering alcoholics) and a brief intervention delivered by a specialist was superior to the specialist intervention alone. This study is unusual in that participants met diagnostic criteria for an alcohol use disorder, and initiating treatment or self-help group involvement and abstinence were focal outcomes. Patients were given usual care, a 5- to 15-minute session of advice from an addiction medicine consultant, or the session of advice in addition to a 30- to 60-minute peer intervention visit from a pair of Alcoholics Anonymous (AA) volunteers. The volunteers had been trained in conveying the AA message and sharing their personal stories. They were matched with the patients on gender and usually on race. Results from the followup at 6 months after discharge indicated that both advice alone and advice plus the peer intervention were superior to usual care in terms of abstinence. Not surprisingly, the combination of advice plus peer intervention was superior to both the advice and the usual care in terms of initiating treatment or self-help.

## Overall Efficacy of Brief Interventions

The overall efficacy of brief interventions, particularly in primary care, has been supported by numerous empirical studies, systematic reviews, and meta-analyses (D’Onofrio and Degutis 2002; Moyer et al. 2001; Whitlock et al. 2004). However, this support is qualified by the fact that the long-term efficacy of these interventions is limited (Wutzke et al. 2002). In addition, their effects may not be as large as previously thought, as shown in a recent meta-analysis that accounted for the influence of study dropouts (Ballesteros et al. 2004). Brief interventions are presumed to be cost-effective, but just a few studies have investigated this. Fleming and colleagues (2002; described previously) concluded that their intervention, which cost $205 per patient, saved $712 in medical costs, $102 in legal costs, and $7,171 in motor vehicle accident costs for each patient, with a net benefit–cost ratio of 39 to 1.

## Technology in the Delivery of Alcohol Screening and Brief Interventions

Many of the challenges to disseminating and implementing brief interventions—such as the time required to deliver them in busy places such as emergency departments and overscheduled environments such as primary care offices, the training required by staff so that they feel comfortable in providing interventions to patients, and the cost of providing interventions—may be mitigated by the use of technology. Computer programs can be used to efficiently screen for alcohol misuse and may encourage participants to provide more honest disclosure. In addition, high-quality, consistent interventions can be delivered via computer (including over the Internet) while providing information tailored to participants’ situations.

For example, the Health Habits Survey, developed by Butler and colleagues (2003), is delivered using a computerized kiosk designed to sit in a physician’s office. This interactive, bilingual survey presents questions in text and voice-over that a user can answer by touching choices on the screen. It can assess the user’s alcohol consumption and stage of readiness to change, and can provide tailored feedback intended to reduce the user’s risky drinking. The user can read the feedback report on the screen or print it out to take home, and may authorize the physician to review the report.

The Drinker’s Check-Up is a computer program based on motivational intervention techniques. This computerized intervention can be used with patients as a lead-in to more formal treatment; it also is helpful for therapists or counselors who do not have experience in treating patients with alcohol-related problems (Squires and Hester 2004). The program uses modules that deliver all the steps a patient would encounter in the course of treatment. The assessment module helps determine whether the user would be considered to be at low, medium, high, or very high risk for excessive drinking and recommends whether the user might benefit from going through the more formal treatment program. The feedback module provides information on the user’s score on each of the assessments and responses to common client reactions to such feedback. The decisionmaking module first allows users to specify their level of readiness to change. Those who are not at all ready have the option of receiving some minimal information before exiting the program. Those who are unsure go through a detailed exercise exploring reasons for and against changing. Those who are at the point where they are ready to change are given a menu of goal options. Once they decide which to pursue, they are taken through exercises to develop a plan of change and are referred to additional resources, such as Web links, self-help groups and materials, and lists of therapists.

Using an innovative interactive, multimedia, video doctor technology, Gerbert and colleagues (2003) developed a patient-centered, supportive, nonjudgmental intervention based on motivational interviewing. A laptop computer program presents several video clips of an actor-portrayed doctor asking health questions and delivering messages. The interpersonal style of the “doctor” is warm, respectful, nonjudgmental, and collaborative. This program employs branching logic that allows users to customize the content of the video clips according to their gender, level of drinking, readiness to change, and desire for information. These messages provide personal feedback, allow users to make their own choices about changing, give gentle recommendations and suggestions for making changes, and foster a sense of self-efficacy among users. Pilot results indicated that, although users reported they would be most comfortable with an in-person consultation with a doctor, they responded positively to the video doctor intervention, and it was accessible even to those with little computer experience.

Computerized assessment and brief interventions hold great promise. Patients can use such programs via the Internet on their home computers, which offers them privacy and does not restrict them to using the programs at a specific time or location. Results from the patients’ assessments can be made accessible to their health care providers for followup during office visits.

## Conclusion

Overall, brief interventions appear to be useful in a variety of settings and are potentially cost-effective in reducing hazardous or harmful alcohol consumption. Medical settings such as emergency departments or trauma centers, in particular, may afford “teachable moments” when people are particularly open to changes in their alcohol use behavior. Computer/Internet technology provides a means for assessing alcohol misuse and implementing brief interventions when time constraints or lack of resources or training in intervention techniques are issues. It remains to be seen, however, if these types of interventions are as effective as those delivered by a live authority figure.

## Footnotes

1 For the purposes of this article, “risky or hazardous drinking” is defined as “more than 7 drinks per week or more than 3 drinks per occasion for women, and more than 14 drinks per week or more than 4 drinks per occasion for men” (U.S. Preventive Services Task Force 2004, p. 554). “Harmful drinking” is defined as “experiencing physical, social, or psychological harm from alcohol use” (U.S. Preventive Services Task Force 2004, p. 554), but without meeting DSM–IV criteria for alcohol abuse and dependence. It should be noted that these definitions vary in different studies.
